# Awareness of Practice and Comparison with Best Evidence in Surgical Site Infection Prevention in Colorectal Surgery

**DOI:** 10.1089/sur.2019.203

**Published:** 2020-03-19

**Authors:** Josep M. Badia, Anna L. Casey, Inés Rubio-Pérez, Nares Arroyo-García, Eloy Espin, Sebastiano Biondo, José M. Balibrea

**Affiliations:** ^1^ Department of Surgery, Hospital General de Granollers, Barcelona, Spain.; ^2^ Universitat Internacional de Catalunya, Barcelona, Spain.; ^3^ Department of Clinical Microbiology, University Hospitals Birmingham NHS Foundation Trust, Birmingham, United Kingdom.; ^4^ Department of Surgery, Hospital Universitario la Paz, Madrid, Spain.; ^5^ Department of Surgery, Hospital Universitari Vall d'Hebrón, Barcelona, Spain.; ^6^ Department of Surgery, Hospital Universitari de Bellvitge, L'Hospitalet de Llobregat, Barcelona, Spain.; ^7^ Department of Surgery, Hospital Clínic de Barcelona, Barcelona, Spain.; ^8^ Universitat de Barcelona, Barcelona, Spain

**Keywords:** colorectal surgery, mechanical bowel preparation, oral antibiotic prophylaxis, prevention, **s**urgical site infection

## Abstract

***Background:*** The use of mechanical bowel preparation and prophylaxis with oral antimicrobial agents can prevent surgical site infection (SSI) in colorectal surgical procedures, but routine adoption of these and other practices by surgeons has been limited. The aim of this study was to determine the actual practice and surgeon beliefs about preventative measures in elective colorectal operations and to compare them with established recommendations.

***Methods:*** Web-based survey was sent to colorectal surgeons assessing knowledge, beliefs, and practices regarding the use of preventative measures for SSI.

***Results:*** Of 355 surgeons, 33% had no feedback of SSI rate; 60% believed in evidence for normothermia, wound edge protection, and use of alcohol solution, and reported use of these strategies. There was a discrepancy in the assumed evidence and use of hyperoxia, glove replacement after anastomosis, surgical tools replacement, and saline surgical site lavage. Most of respondents believe that oral antibiotic prophylaxis diminishes infection, but is indicated only by one third of them. Few surgeons believe in MBP, but many actually use it. Most surgeons believe that there is a discrepancy between published guidelines and actual clinical practice. As proper means to implement guidelines, checklists, standardized orders, surveillance, feedback of SSI rates, and educational programs are rated most highly by surgeons, but few of these are in place at their institutions.

***Conclusions:*** Gaps in the translation of evidence into practice remain in the prevention of SSI in colorectal surgical procedures. Several areas for improvement have been identified. Specific implementation strategies should be addressed in colorectal units.

Surgical site infections (SSI) have now become the most common hospital-acquired infection in Europe (21.6%) [1] and the most frequent complication after surgical procedures. Indeed, rates of up to 20% have been reported for colorectal operations [2,3]. A significant financial burden, prolonged hospitalization, and higher consumption of antibiotic agents are associated with SSI in colorectal surgical procedures [4,5].

The multitude of preventive measures has demonstrated varying levels of efficacy and adoption [6]. Some measures, such as mechanical bowel preparation (MBP) and oral antibiotic prophylaxis, are specific for colorectal operations and are used irregularly [7]. A gap may exist between the best scientific evidence and clinical practice as far as SSI avoidance in colorectal surgical procedures are concerned. There is a wide consensus that antibiotic agents must be used before colorectal operation, but it is still debated whether they should be administered exclusively by an intravenous route or by a combined route with oral non-absorbable antimicrobial agents.

Thus, the role of bowel preparation and the opportunity to combine it with oral antibiotic agents have been discussed extensively. In recent decades, numerous published studies fueled this debate and assessed the indication of oral antimicrobial agents and MBP in patients scheduled for elective colorectal procedures. Determining the level of adoption of the preventive measures is vital before development of dissemination policies and generating bundles.

The aim of this study was to undertake a survey among colorectal surgeons to determine their level of knowledge of prevention strategies, the preferences, and the actual adoption of the measures in their hospital. Questions included the use of MBP (alone or combined with oral antibiotic agents), systemic antibiotic prophylaxis, patient skin preparation, surgical site edge protection, normothermia, peri-operative hyperoxia, and surgical instrument replacement.

## Methods

A Web-based survey (SurveyMonkey^©^; https://es.surveymonkey.com/r/FBVR59C) was distributed to the members of the Spanish Association of Coloproctology and the Colorectal Chapter of the Spanish Association of Surgeons. A link to the site page containing the survey was distributed via e-mail, newsletter, and Twitter. The survey was open for 52 days (January 8 to February 28, 2018).

A board from the Surgical Infection Section led a review of the literature to be utilized in the assessment of the results. The most recent recommendations from clinical practice guidelines were analyzed [8–15]. A summary is shown in Table 1. Further, an extensive review of specific measures for colorectal operations was used for evaluation [16]. As a result of the review, the preventive measures analyzed were distributed into two groups: high or low level of evidence (Table 2).

**Table 1.** Summary of Recommendations from National and International Clinical Practice Guidelines

Preventive measure	NICE^a^ [9,10] (2008, 2017)	Spanish [11] 2010	Canadian [12] 2014	Anderson [13]	Scotland [14] 2015	Allegranzi [7] (WHO)^c^ 2016	Berrios-Torres [8] (CDC)^d^ 2017
Hair removal	Do not	Do not	Do not	Do not	Do not	Do not	Do not
	(if YES: clipping)	(if YES: clipping)	(if YES: clipping)	(if YES: clipping)	(if YES: clipping)	(if YES: clipping)	(if YES: clipping)
Oral antibiotic prophylaxis						Yes	
Mechanical bowel preparation	Do not	Do not				Yes	
Antiseptic skin preparation	Aqueous or alcohol-based PI or CHG	PI or CHG	Alcohol CHG > PI	Alcohol PI or CHG	Alcohol CHG > PI	Alcohol CHG	Alcohol
Plastic incise drapes	Do not (if YES: iodophor-impregnated)	Do not		Do not		Do not	Do not
Surgical site edge protection				Yes, plastic (dual>single)		Yes	
Normothermy	Yes	Yes	Yes	Yes	Yes	Yes	Yes
Oxygenation	Yes (maintain O_2_ sat >95 %)	“Sufficient perfusion”		Yes Supplemental O_2_	Yes (maintain O_2_ sat >95 %)	Yes Supplemental O_2_	Unresolved
Surgical site irrigation	Do not	Do not				Unresolved	Yes (PI solution)
Antimicrobial suture			Do not	Do not		Yes	Yes
Negative pressure surgical site therapy						Yes (high risk)	

Blank: No recommendation issued.

^a^ NICE = National Institute for Health and Care Excellence.

^b^ SHEA/IDSA = Society for Healthcare Epidemiology of America/Infectious Diseases Society of America.

^c^ WHO = World Health Organization.

^d^ CDC = Centers for Disease Control and Prevention.

Different systems of evidence quality gradation are used. These recommendations are also supported by different levels of evidence. Modified from Badia JM et al.[6]

PI = povidone iodine; CHG = chlorhexidine gluconate.

**Table 2.** Summary of Questions and Classification of Evidence Based on the Expert Group Evaluation

	Question	
**1.**	**Demography**	
	Years of experience	
	Existence of a Colorectal Unit in the hospital	
	Knowledge of the SSI rate in colorectal surgery of the unit/hospital	
	Existence of an enhanced recovery after surgery (ERAS) program in the unit/hospital	
	Number of colectomies in unit/hospital per year (<100, 100–200, >200)	
**2.**	**Pre-operative measures**	**Level of evidence**
	Mechanical bowel preparation	High
	Prophylaxis with oral antibiotic agents	High
	Hair removal	High
	Use of electrical clipper if hair removal	High
	Alcoholic solutions for patient skin antisepsis	High
**3.**	**Intra-operative measures**	
	Normothermia	High
	Glucose control	High
	Hyperoxia	Under evaluation
	Plastic surgical site-edge protection devices	High
	Peritoneal lavage at the end of laparotomy	Low
	Antiseptic coated sutures	Low
	Policy on intra-operative changes of gloves	Low
	Policy on replacement of surgical instruments prior to closing incision	Low
	Sugical site lavage before closure	Low
	Negative pressure surgical site therapy	Low

On each question, they were asked about whether evidence supports method and the actual use of it.

The survey aimed to evaluate the knowledge, opinions, and practices of colorectal surgeons on preventive measures including MBP, oral antibiotic prophylaxis, and the use of drains. Further, the questions addressed the level of accordance between their beliefs and the protocols or the usual practice of their units. The agreement rate between the beliefs and usual practice of all respondents was calculated on a scale from 0 to 100.

For discussion, the answers on the use of major SSI preventive measures were contrasted with the suggestions of the expert group. Other questions were related to the types of complications that colorectal specialists believe are avoided by the use of oral antimicrobial prophylaxis or MBP, reasons for not using either of them, type of oral and systemic antibiotic agents and cathartics used, and those policies already in place or that should be introduced to reduce SSI at respondents' hospitals.

The project was registered with the ClinicalTrials.gov Identifier: NCT03883399 and has been reported in line with the Consolidated Criteria for Reporting Qualitative Research criteria. The outcomes are shown in percentages on the total responses attained. Responses were introduced into a computer database that was analyzed using the SPSS program (v.10.0, Chicago, IL). The results are analyzed using the chi-square test with statistical significance defined as p < 0.05.

## Results

Three hundred and fifty-five responses were obtained from 654 surgeons (54.3% response rate). Demographics of the respondents are given in Table 3. Most of the respondent surgeons practice in a high workload colorectal unit. Higher number of colectomies per year correlated with the existence of a colorectal surgery unit (33.7% in low volume vs. 98.7% in high volume) and the existence of a departmental enhanced recovery after surgery (ERAS) program for colorectal operation (40.9% in low volume vs. 68.9% in high volume).

**Table 3.** Demographic Details of Respondents and Centers

Years of experience of responding surgeons	<10 y	>11 y
	165/351 (47%)	255/351 (53%)
Type of hospital	Private	Public
	10/220 (4.5%)	210/220 (95.5%)
Type of hospital	Primary	Tertiary/University
	87/351 (24.8%)	264/351 (75.2%)
Annual activity of the Colorectal Unit	< 100 colectomies	> 101 colectomies
	91/351 (25.9%)	260/351 (74.1%)
Is there a Colorectal Surgery Unit in your hospital?	Yes	No
	258/351 (73.5%)	93/351 (26.5%)
Do you periodically have feedback on the SSI rate of your hospital/unit?	Yes	No
	235/351 (67%)	116/351 (33%)
Do you have a departmental ERAS protocol for colorectal surgery?	Yes	No
	218/351 (62.1%)	133/351 (37.9%)

SSI = surgical site infection; ERAS = enhanced recovery after surgery.

It is noteworthy that only 67% of responding surgeons have periodic feedback about the SSI rate of colorectal cases. Even in high-volume units (>200 colectomies per year), only 73.3% of surgeons are aware of their SSI rate. Surgeons working within a colorectal unit have more feedback (74.5% vs. 43.3%; p = 0.05) and are more likely to run an ERAS program (73.4% vs. 30.4%) than those who do not. More than 73% of respondents consider that a mechanism to provide specific feedback to surgeons should be implemented, but only 39% stated that it was currently in place in their hospitals.

The actual level of use of preventive measures is summarized in Table 4. More than 60% of respondents indicated that there was high evidence for hair clipping, use of alcohol solution for antiseptic skin preparation, and maintenance of normothermia, and stated use of these policies. Antiseptic coated sutures and negative pressure therapy on the closed surgical site are scarcely used (6.2% and 7.1%, respectively), in accordance with the respondents' knowledge that there is poor evidence to support these strategies (13.4% and 12.1%).

**Table 4.** Actual Use of Surgical Site Infection Prevention Measures

Pre-operative prevention measures	Yes	No
Oral antibiotic agents	78/239 (32.6%)	161/239 (67.4%)
Mechanical bowel preparation (any kind/any site)	231/240 (96.2%)	9/240 (3.8%)
Bowel preparation at patient's home	90/224 (40.2%)	134/224 (59.8%)
Hair removal	178/231 (77.1%)	53/231 (22.9%)
Hair removal with clipper	161/237 (67.9%)	76/237 (32.1%)

Intra-operative prevention measures		
Alcohol solution for skin antisepsis	156/234 (66.6%)	78/234 (33.3%)
Normothermia	166/238 (69.7%)	72/238 (30.3%)
Hyperoxia 80%	71/220 (32.3%)	149/220 (67.7%)
Glucose control	131/227 (57.7%)	96/227 (42.3%)
Glove exchange at end of anastomosis	174/234 (74.4%)	60/234 (25.6%)
Glove exchange at the end of laparotomy	211/229 (92.1%)	18/229 (7.8%)
Peritoneal lavage at the end of laparotomy	176/229 (76.9%)	53/229 (23.1%)
Antiseptic coated sutures	14/225 (6.2%)	211/225 (93.8%)
Replacement of surgical instruments prior to closing incision	154/233 (69.1%)	79/233 (33.9%)
Surgical site lavage prior to closure	197/229 (86%)	32/229 (14.0%)
Negative pressure surgical site therapy	16/224 (7.1%)	208/224 (92.9%)

**FIG. 1.**
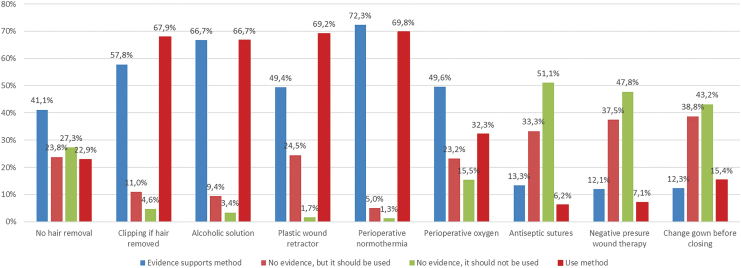
Infection prevention measures that demonstrated good concordance (less than 20% of difference) between surgeons' beliefs regarding evidence (positive or negative) and clinical practice patterns. Color image is available online.

**FIG. 2.**
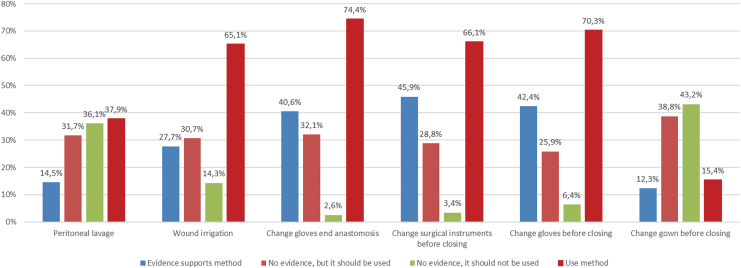
Infection prevention measures that demonstrated discordance (more than 20% of difference) between surgeons' beliefs regarding evidence and clinical practice patterns. Color image is available online.

There is a group of measures that shows good concordance (less than 20% of difference) between surgeons' beliefs (positive or negative) and their actual practice (Fig. 1), all but omission of hair removal in accordance with the study group recommendations. There was a disagreement in the supposed level of evidence and the self-reported use of other measures, such as glove replacement after finishing the anastomosis (40.6% vs. 74.4%), surgical tool replacement before closing the incision (45.9% vs. 66.1%), and surgical site irrigation before closing the skin (27.7% vs. 65.1%). Figure 2 shows this group of methods with a higher level of disagreement, most of them being measures with low level of evidence, although commonly implemented in operating theaters.

**FIG. 3.**
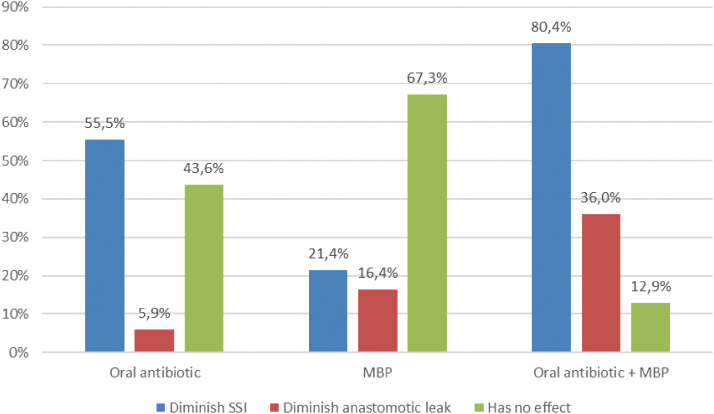
Surgeons' beliefs about oral antibiotic prophylaxis and mechanical bowel preparation (MBP) efficacy. Color image is available online.

**FIG. 4.**
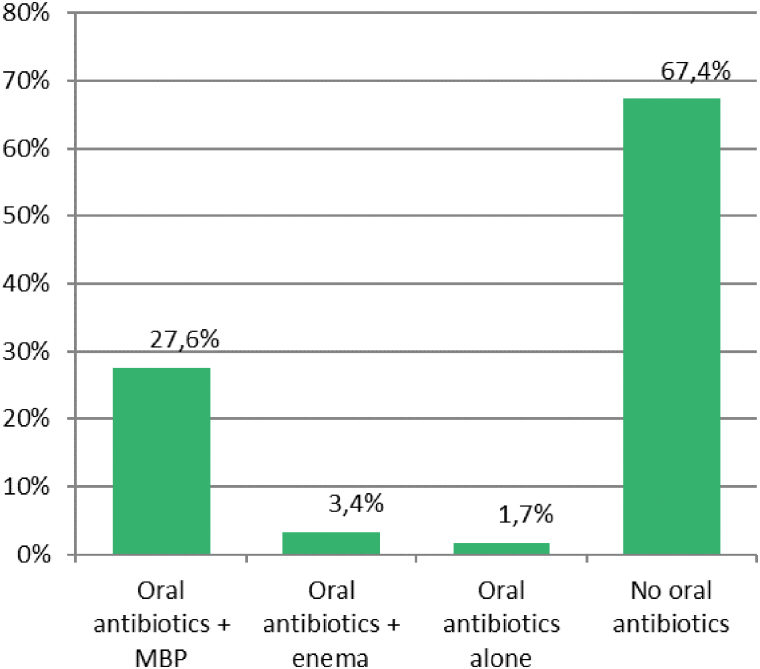
Actual use of oral antibiotic prophylaxis of responding surgeons. MBP = mechanical bowel preparation). Color image is available online.

Regarding oral antibiotic prophylaxis, most of respondents believe that this measure reduces the risk of SSI, either alone (55.5%) or in combination with MBP (80.4%) (Fig. 3), but it is prescribed only by 32.6% of surgeons, mostly in combination with MBP (27.6%) (Fig. 4). There were no statistical differences in beliefs or clinical practice about MBP and oral antibiotic agents among surgeons belonging to high volume or low volume units, or working in hospitals with or without colorectal units. The most common reason why oral antibiotic agents are not used is that they are not included in the hospital protocols (65.5%). Only 8.2% of responders believe oral antibiotic agents are not useful.

**FIG. 5.**
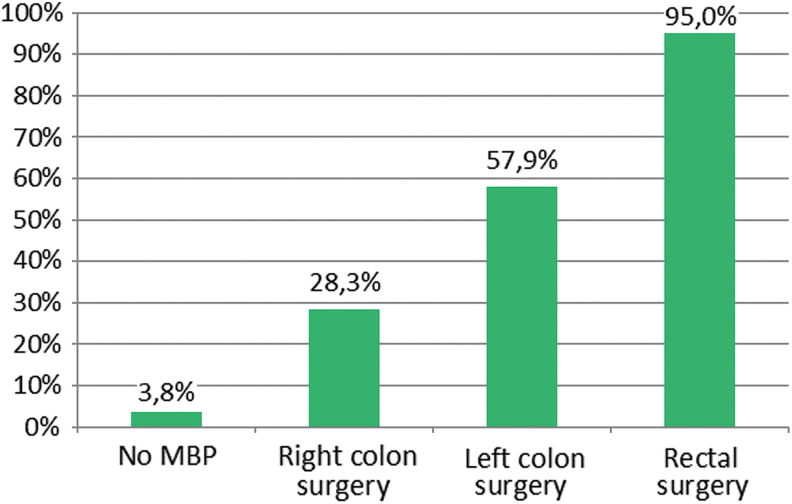
Actual use of mechanical bowel preparation (MBP) of responding surgeons. Color image is available online.

Mechanical bowel preparation is believed to be beneficial in all colorectal cases by 14.6% and only in rectal surgical procedures by 67.9% of respondents, but is actually used in 86.3% and 95%, respectively (Fig. 5). The accordance rate for oral antibiotic agents and MBP use had an average of 67.8% and 76.0%, respectively. The techniques and products used for MBP are shown in Table 5. **The** most used antibiotic agents for systemic prophylaxis were amoxicillin-clavulanate (41.8%) or a combination of cephalosporin plus metronidazole (40.2%). Ertapenem was not used for prophylaxis.

**Table 5.** Methods/Products Used for Mechanical Bowel Preparation

Pre-operative fiber-free diet (3–5 d)	179/241 (74.3%)
Enema bowel cleanse	50/228 (21.9%)
Anterograde mechanical bowel preparation	161/228 (70.6%)
Polyethylene glycol based	91/219 (41.6%)
Phosphate based	44/219 (20.1%)
Both (polyethylene glycol/phosphate)	71/219 (32.4%)

**FIG. 6.**
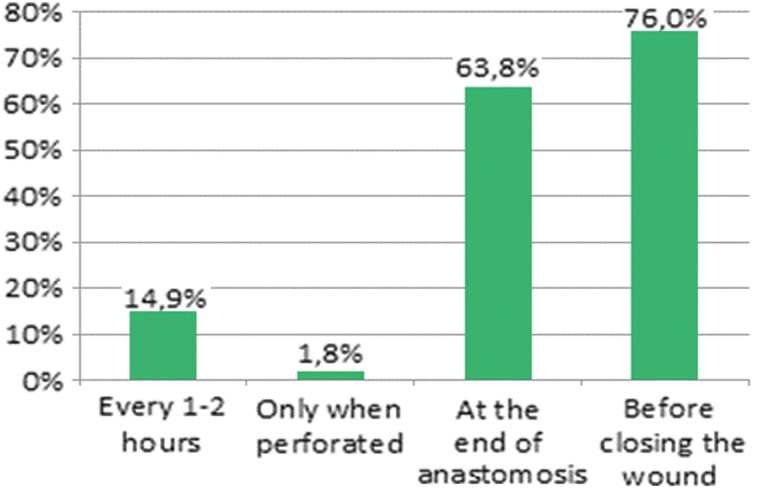
Intra-operative replacement of gloves. Color image is available online.

**FIG. 7.**
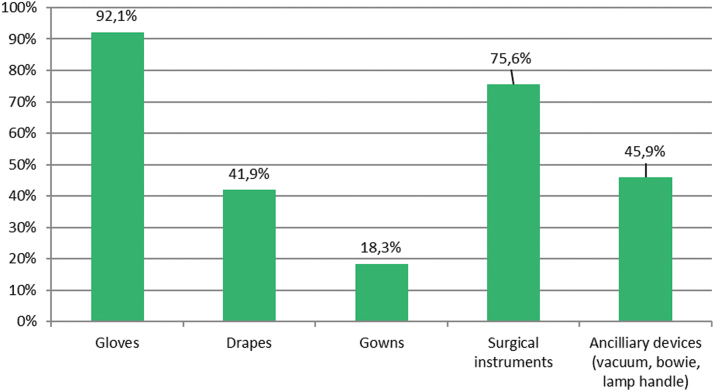
Surgical instrument replacement. Color image is available online.

The intra-operative policy on gloves and surgical device replacement are shown in Figures 6 and 7. Only 20.1% of surgeons state that they do not leave abdominal drains in situ after colorectal surgical procedures. Conversely, drains are used in right colectomy (24%), left colectomy (41%), and rectal operations (75.5%). Respondents working within colorectal units use ambulatory MBP and peri-operative glucose control more frequently. On the contrary, they use less abdominal drains and practice less peritoneal irrigation after operation.

**FIG. 8.**
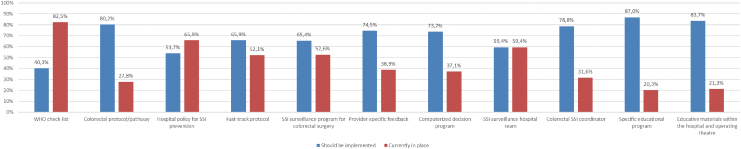
Proportion of surgeons who believe the prevention strategies should be used and comparison with their actual implementation at their hospitals. WHO = World Health Organization; SSI = surgical site infection. Color image is available online.

Most of surgeons believed that it was a discrepancy between published guidelines and actual clinical practice (70% of overall disagreement rate). To reduce SSI rates, the World Health Organization's checklists, standardized orders, surveillance, and educational programs and feedback were the most highly rated strategies by surgeons, but few of these were in place at their institutions (Fig. 8). More than 70% of surgeons believed that a colorectal protocol or pathway, computerized decision programs, and specific educational programs on SSI prevention in colorectal operations could be helpful, but less than a third stated that these approaches were already in place at their hospitals. Surgeons working in colorectal units gave more importance to feedback of SSI rates and to the establishment of ERAS programs than the rest of respondents.

## Discussion

Regardless of the publication of several clinical guidelines for the prevention of SSI during the last decade, it seems that compliance with passively disseminated policies should improve [6]. This study focused on the degree of utilization of the key preventive measures for post-operative infection in colorectal surgical procedures. This could be used to design and disseminate specific bundles of measures to prevent SSI in this high-risk type of operation. The detected low rate of feedback to surgeons on the SSI rate is worrisome: Although most of the responders work in high-volume colorectal units, a quarter of them are not aware of their SSI rate. In several studies, surveillance of SSI with confidential feedback to surgeons has been found to reduce the risk of SSI, also in colorectal operations [17].

The survey shows a wide variability in the use of some of the preventive methods. Likewise, a gap between scientific evidence and clinical practice has been detected in the prevention of SSI in elective colorectal surgical procedures. Skin antisepsis with alcoholic solutions, the use of a plastic wound retractor, and maintenance of normothermia are the processes with a high level of concordance of responding surgeons to the endorsements of up-to-date practice guidelines. Other strategies with low level of evidence, such as peritoneal and surgical site irrigation with sodium chloride are nevertheless often used, perhaps by surgical tradition and belief that these areas have been insufficiently evaluated to omit their use.

On the contrary, different actions with a high grade of recommendation show a low level of “real life” application. Further, a few practices that are not endorsed by guidelines or that are even recognized to raise the SSI rate are continued. The main identified areas to address are the high level of razor shaving and the low use of intra-operative glove changes.

Probably the most controversial measures in the field of colorectal surgery are the role of MBP and prophylactic oral antimicrobial agents in the prevention of SSI. The lack of evidence to demonstrate the efficacy of MBP to reduce rates of SSI, its potential adverse effects, and the emergence of multi-modal rehabilitation programs have prompted a decrease in its utilization. In 2017, a European survey showed an oral prophylaxis use of only 11% and a routine use of MBP of 29.6% [7].

The expert panel for the present study concluded, however, that clinical trials show a decrease in SSI when prophylaxis with oral antibiotic agents is combined with MBP [16]. The data generated by randomized and observational trials suggest that oral antibiotic prophylaxis in association with MBP plays a pivotal role in decreasing the risk of all kinds of SSI, anastomotic leak, post-operative ileus, readmissions, and death, without being related to an augmented risk of *Clostridium difficile* infection.

In our study, few responding surgeons believe in the MBP efficacy to prevent SSI or dehiscence, but many use it in their units. Conversely, many of them believe in the benefits of oral antibiotic prophylaxis, but few of them utilize it. This reflects the debate that still exists among colorectal surgeons.

This debate is fueled by the scientific literature, where several measures to reduce SSI in colorectal operations have been evaluated. Some measures have been assessed in controlled trials, some with conflicting findings, while others are the consequence of clinical observation or everyday surgical practice and can hardly be subjected to well-thought-out methodic analysis.

In addition, the recommendations of practice guidelines, albeit founded on the same original findings, not infrequently reach different conclusions, probably from several causes: Not all preventive measures have been investigated adequately; there is inconsistency in the selection of clinical studies in systematic reviews and the diverse systems of quality gradation that are used. Maybe this is the reason why the level of awareness and use of the acknowledged measures for the prevention of SSI have shown great erraticism, and compliance with published guidelines has been described as low [18], even in high-risk surgical specialties such as colorectal surgery [19].

The literature on knowledge translation warns of difficulties related to uptake and compliance with guidelines [20]. Further, some studies previously have shown a substantial gap between the best scientific evidence and clinical practice as far as SSI prevention is concerned [6,21]. These results show that many senior surgeons and trainees fail to implement the best surgical practices despite the awareness of evidence supporting them, especially with regard to use of oral antibiotic prophylaxis and MBP.

Most of our responding surgeons are aware of the difficulties in knowledge translation to surgical practice. They believe that specific colorectal pathways, fast-track protocols, provider feedback, educational programs, and computerized decision programs are important when making decisions regarding prevention of SSI, although few of them are in practice in their organizations.

According to the Normalization Process Theory [22], patients, professionals, managers, and policy-makers face two relevant types of difficulties as they attempt to get advancements into practice: Process issues (about the utilization of novel perspectives, acting and organizing in healthcare) and organizational issues (about the incorporation of new schemes of practice into existing hierarchical and qualified sceneries). Normalization Process Theory is a descriptive model that may help researchers and clinicians understand these procedures, and perhaps may facilitate the introduction of multi-faceted processes and new technologies in health systems [23].

A few surveys have been published on this subject, most of them evaluating specific procedures [24–26], or in general surgical procedures [6]. One survey on colorectal operations covered only a small geographic area [21]. The present survey is to date the only one in particular that gathers input from colorectal specialists at a national level and the one that acquires the highest absolute amount of responses.

### Limitations of the study

This study has several limitations. First, the response rate was 54.3%, although it is difficult to calculate it accurately, given the doubt about the amount of colorectal surgeons who in fact received the study information. The absolute quantity of surgeons who have responded is high, however. Online surveys are able to get a big number of responses maybe at the expense of obtaining a low percentage of response. In spite of this limitation, we consider that there is a well-adjusted representation of diverse categories of centers, which suggests that the findings can be extended to the actual surgical practice.

The work may also be limited by self-report bias; because self-reporting has been shown to overrate performance [27].

## Conclusions

Our results suggest that gaps in the translation of best evidence into actual practice in the prevention of SSI in colorectal surgical procedures are persistent, even within academic environments. Some areas of improvement have been detected and should be addressed specifically in colorectal units.

The active diffusion of homogenous SSI prophylactic recommendations with a high rate of scientific evidence ought to decrease taxes of SSI consistently. Implementation policies must concentrate not only on the professionals, but also on the settings in which they practice. Understanding the level of implementation of preventative measures and the level of awareness of the providers on the available scientific evidence is crucial.
